# A Rare Presentation of Synchronous Colorectal Adenocarcinoma

**DOI:** 10.7759/cureus.47337

**Published:** 2023-10-19

**Authors:** Arwa Battah, Iyad Farouji, Theodore R DaCosta, Byron Okwesili, Abdelhadi Farouji, Reshma John, Daphne Gonzalez, Saraswathi Lakkasani, Yatinder Bains

**Affiliations:** 1 Internal Medicine, Saint Michael's Medical Center, Newark, USA; 2 Gastroenterology and Hepatology, Saint Michael's Medical Center, Newark, USA; 3 Internal Medicine, St. George's University School of Medicine, St. George's, USA; 4 Gastroenterology, Saint Michael's Medical Center, Newark, USA

**Keywords:** laparascopic surgery, oncology surgery, gi oncology, synchronus colorectal cancer, adnocarcinoma

## Abstract

Synchronous carcinoma is defined as multiple malignant lesions presented in a single patient at initial diagnosis. Synchronous colorectal adenocarcinoma is a rare entity that has been increasingly recognized, likely due to the significant improvement in imaging and diagnostic tools. Making the appropriate diagnosis of synchronous colorectal cancer has a major role in the management's determination and treatment plans. Herein, we are reporting a case of a 73-year-old gentleman who was diagnosed with synchronous colorectal adenocarcinoma with two masses in the left colon and was treated initially surgically followed by chemotherapy.

## Introduction

Although colorectal cancer (CRC) is one of the most common cancers in the United States, synchronous CRCs are less common and can complicate the treatment plan. Synchronous CRC is diagnosed when two or more primary colorectal lesions are diagnosed simultaneously or within six months of initial diagnosis [1]. Synchronous lesions are more commonly seen in the right colon; however, in our case, the patient presented with two left-sided colon lesions. Direct visualization with colonoscopy can be further complicated by obstructing lesions and synchronous lesions can potentially be missed requiring post-resection colonoscopy and potentially another resection [2]. Surgical treatment for patients with synchronous lesions further complicates the treatment plan. Some studies recommend subtotal colectomy and others recommend appropriate surgical resection with follow-up colonoscopy [2]. Prompt diagnosis of synchronous colorectal lesions can provide physicians with better insight regarding the patient’s treatment plan and potentially prevent repeat surgical resections after initial treatment. 

## Case presentation

A 73-year-old gentleman with a past medical history of heart failure with preserved ejection fraction, moderate to severe aortic stenosis, hypertension, diabetes mellitus type 2, history of stroke with left-sided residual weakness presented to the emergency department for one month of worsening low back pain. The pain began worsening with standing for the past month and radiated down his left thigh. He also began experiencing difficulty ambulating. He was able to ambulate without help prior. He complained of flares of on-and-off back pain over the past 20 years but never had it evaluated. All other reviews of systems were unremarkable. He was a former smoker, quit 50 years ago, and drank alcohol socially.

Initial physical examination, the patient was normothermic, slightly hypertensive, and tachycardic. Mild discomfort due to back pain. He also had a left-sided facial droop. Labs showed elevated serum glucose and urobilinogen. All other lab results were within normal limits.

**Figure 1:**
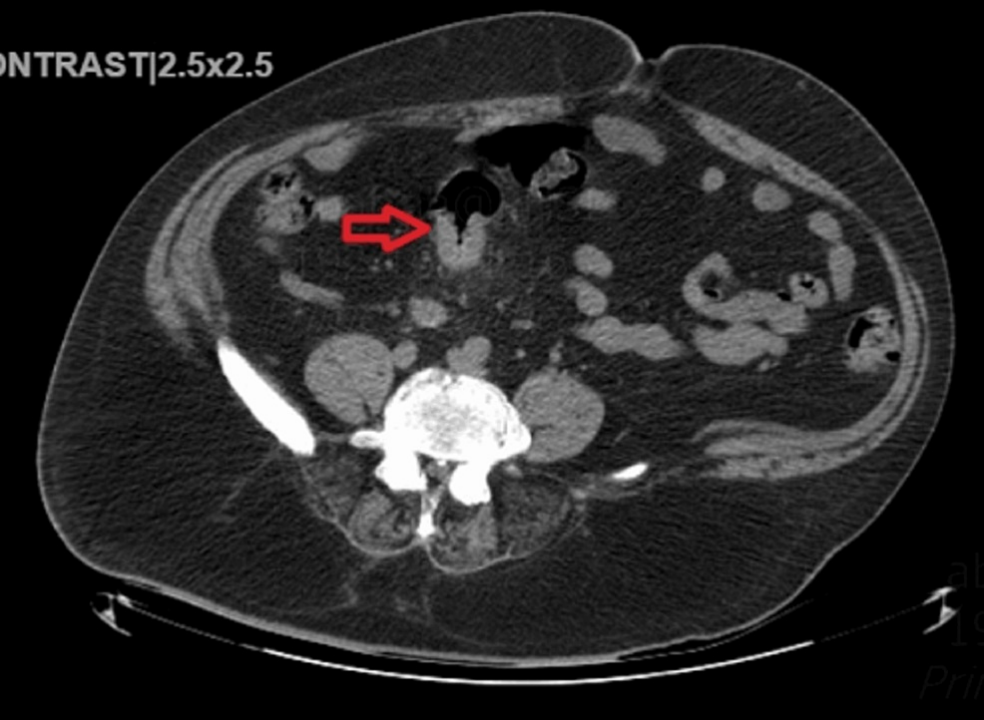
CT abdomen revealed circumferential wall thickening of the sigmoid colon (red arrow).

**Figure 2:**
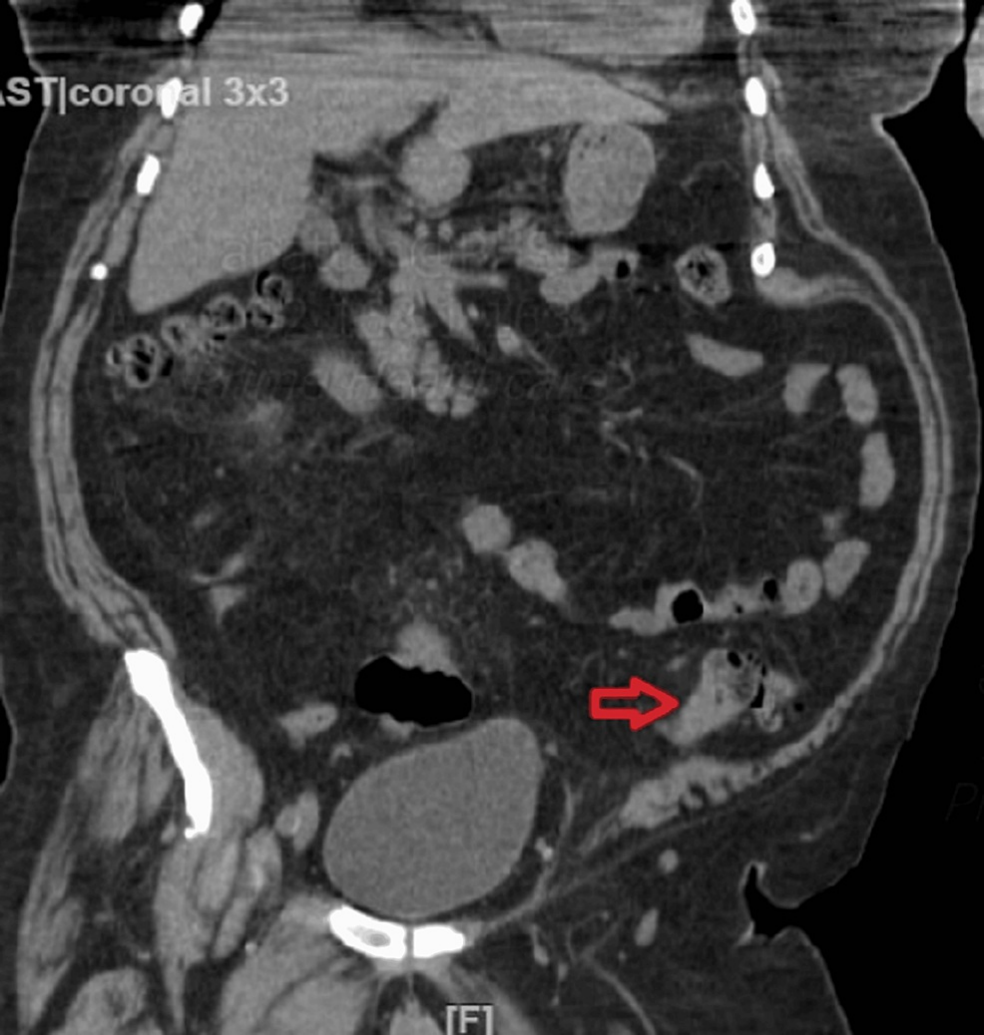
CT abdomen, coronal section, revealed concentric wall thickness of the descending colon (red arrow).

**Figure 3:**
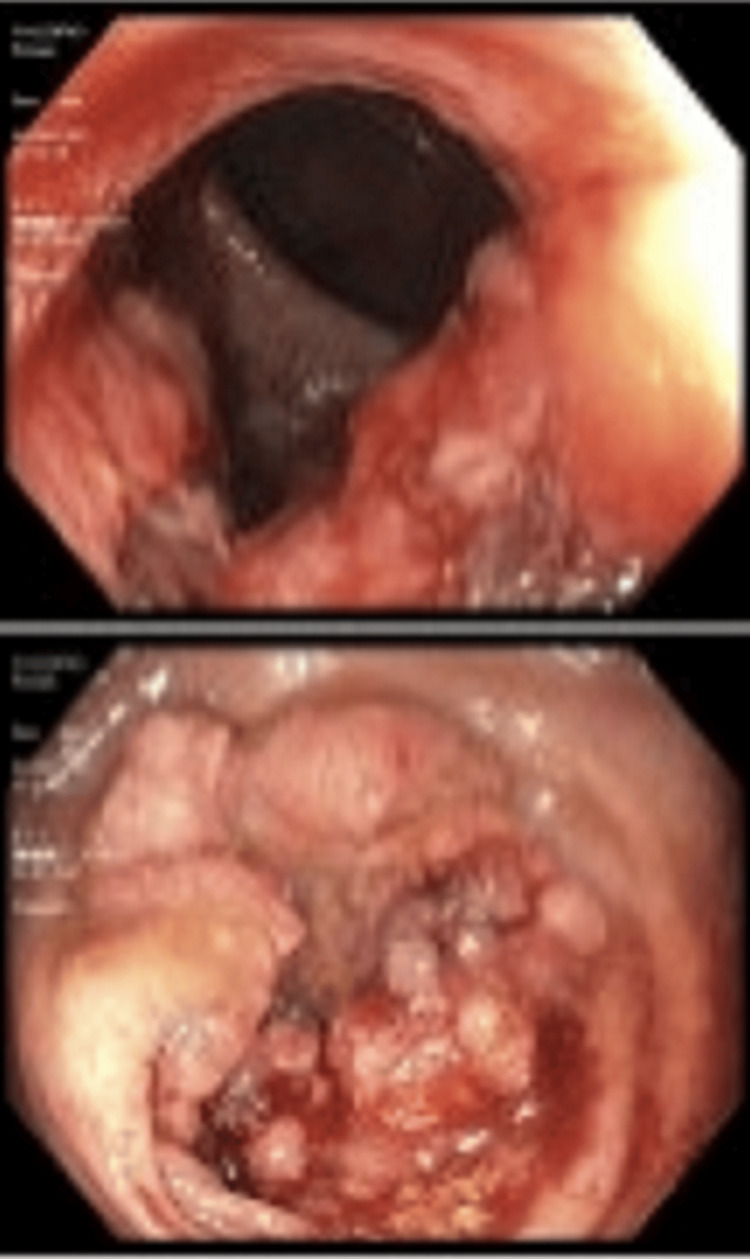
(Up and down) Colonoscopy revealed a fungating mass in the sigmoid colon.

A computerized tomography (CT) of the abdomen/pelvis, showed a segment of focal mural thickening and narrowing of the sigmoid colon over a 5 cm region (Figures 1, 2), colonic diverticulosis without diverticulitis. CT and magnetic resonance imaging (MRI) of the spine revealed multilevel degenerative changes in the lumbar spine. The gastroenterology service was consulted to further evaluate the CT findings. Serum CEA level was 2.4 ng/mL. A colonoscopy was performed which revealed two large fungating, infiltrative polypoid masses (Figure 3). One mass in the descending colon was 45 cm from the anal verge, which was partially obstructed, and the other mass in the sigmoid colon was 25 cm from the anal verge, which was non-obstructing. They took biopsies with cold forceps. In addition, two multilobulated, semi-sessile, and semi-pedunculated 8-10 mm polyps were found in the sigmoid, close to the sigmoid mass, and grade 1 non-bleeding internal hemorrhoids were seen on retroflexion. Because of the partial obstruction of the mass in the descending colon, the proximal colon was not properly visualized during colonoscopy. Positron emission tomography-CT (PET-CT) did not show any evidence of metastatic lesions.

Biopsy results indicated a moderately well-differentiated adenocarcinoma. He was then evaluated by surgery and a left hemicolectomy was performed. The two colonic masses were removed and a colorectal anastomosis was created between the remaining descending colon and rectum with adequate perfusion. 

The immediate postoperative period was unremarkable, and the patient was transferred to the recovery unit. Laboratory results post-procedure were unremarkable and there were no indications of infection or sepsis. Postoperative day 1, the patient was hemodynamically stable and complained of mild post-surgical abdominal pain. Oncology was consulted, the patient was started on post-operative treatment and the patient was instructed to undergo a follow-up colonoscopy in three to six months for proper visualization of the colon proximal to the obstructed descending colon, but he moved to another country, and we lost follow-up. 

## Discussion

Colon cancer is considered one of the most common malignancies in the United States. Several risk factors have been identified including genetics, aging, and environmental factors. In the Western world, most CRCs are sporadic and believed to be caused by environmental factors [3]. Potentially modifiable risk factors, including obesity, diabetes, tobacco use, excessive alcohol consumption, excessive consumption of processed meat and lack of physical activity have been identified consistently throughout observational studies [4]. Of note, patients under 50 years of age presenting with colorectal carcinoma have been associated with genetic hereditary disorders such as familial adenomatous polyposis (FAP) and hereditary non-polyposis colon cancer (HNPCC) [3].

Although colon cancer is considered to be one of the most common malignancies in the United States, synchronous CRC complicates the treatment plan and the potential risk of requiring further surgical treatment. In the context of CRC, synchronous cancer refers to the occurrence of more than one primary cancer in a single patient, either detected simultaneously or within six months of the initial diagnosis. The reported frequency of synchronous CRCs is around 3.5% [5], usually presented as gastrointestinal symptoms. In our case, the patient was only complaining of back pain and there was no evidence of metastasis in the spine.

Those with conditions such as inflammatory bowel diseases (IBD) (ulcerative colitis and Crohn's disease), hereditary nonpolyposis CRC, FAP, and serrated polyps/hyperplastic polyposis are known to be at a higher risk for developing synchronous colorectal carcinoma [6]. These underlying factors contribute to slightly over 10% of cases of synchronous colorectal carcinomas.

Patients with synchronous colorectal carcinoma have a higher proportion of microsatellite instability than patients with solitary colorectal carcinoma. These synchronous colorectal carcinomas often have different patterns of microsatellite instability status, p53 mutation, and K-ras mutation [7].

Concurrent diagnosis of synchronous malignancies is important for the treatment plan and prevents reoperation for advanced metachronous cancers if it is initially missed [8]. Unlike our case, where the patient had two synchronous lesions in the left colon, most studies have reported that the most frequent location for synchronous cancers is the right colon [9]. The rising diagnosis of synchronous cancer can be attributed mainly to advancements in diagnostic techniques like colonoscopy and computed tomography (CT) colonography [10].

The optimal surgical approach for patients with synchronous CRC remains uncertain. Some experts advocate for total or subtotal colectomy as a treatment option [10]. Extensive surgery is typically warranted for patients with synchronous CRC who have known predisposing factors such as FAP, ulcerative colitis, or HNPCC [7]. In other cases, the recommended course of action involves appropriate surgical resection coupled with colonoscopic follow-up examinations. An alternative surgical approach involves the implementation of intraoperative colonoscopy, a method designed for the precise localization of early mucosal tumors and the identification of lesions within the proximal unexamined colon, particularly applicable to cases of obstructive left-sided cancer. In contrast to the conventional postoperative colonoscopy, as employed in our case, intraoperative colonoscopy offers distinct advantages. By providing immediate and detailed visualization of the lesion during the surgery itself, this approach enhances our ability to accurately assess the extent of the pathology [11]. If one of the synchronous cancers is in an early stage, colonoscopic resection (utilizing techniques like endoscopic mucosal resection or endoscopic submucosal dissection) may suffice. However, if the synchronous cancers are widely separated and in advanced stages, dual colon resection might be necessary. Depending on available resources, long-term clinical follow-up may be advisable for some patients diagnosed with synchronous colorectal carcinoma [12].

The prognosis of synchronous CRC is variable. Some studies showed better prognosis in patients with solitary lesions while others had worse outcomes [13]. The prognosis of patients with colorectal carcinoma is influenced by numerous factors, and the presence of synchronous colorectal carcinoma alone may not be a strong independent predictor of survival rates. This case is a rare and unusual presentation of synchronous adenocarcinoma of the left colon, where lower back pain was the only symptom at the time of the presentation.

## Conclusions

Synchronous CRC lesions, although uncommon, increase the mortality compared to solitary lesions. Diagnosis can be further complicated by obstructing lesions preventing passage of the colonoscope to proximal portions of the colon via direct visualization. Thus, a combination of CT scan and colonoscopy is important in early diagnosis of CRC. Ruling out other lesions or polyps via direct visualization through detailed colonoscopy is very important for optimal treatment and surgical plan. This is especially important in high-risk groups such as men, elderly patients, and those with a family history of CRC or IBD.
